# Methodology of Pulsed Photoacoustics and Its Application to Probe Photosystems and Receptors

**DOI:** 10.3390/s100605642

**Published:** 2010-06-03

**Authors:** Harvey J.M. Hou, Thomas P. Sakmar

**Affiliations:** 1 Department of Chemistry and Biochemistry, University of Massachusetts Dartmouth, North Dartmouth, MA 02747, USA; 2 Laboratory of Molecular Biology and Biochemistry, Rockefeller University, 1230 York Avenue, New York, NY 10065, USA

**Keywords:** receptor, photoactivation, rhodopsin, photosynthesis, photoacoustics

## Abstract

We review recent advances in the methodology of pulsed time-resolved photoacoustics and its application to studies of photosynthetic reaction centers and membrane receptors such as the G protein-coupled receptor rhodopsin. The experimental parameters accessible to photoacoustics include molecular volume change and photoreaction enthalpy change. Light-driven volume change secondary to protein conformational changes or electrostriction is directly related to the photoreaction and thus can be a useful measurement of activity and function. The enthalpy changes of the photochemical reactions observed can be measured directly by photoacoustics. With the measurement of enthalpy change, the reaction entropy can also be calculated when free energy is known. Dissecting the free energy of a photoreaction into enthalpic and entropic components may provide critical information about photoactivation mechanisms of photosystems and photoreceptors. The potential limitations and future applications of time-resolved photoacoustics are also discussed.

## 1. Introduction

Pulsed time-resolved photoacoustics provides an unique way to probe the molecular mechanism of electron transfer and proton transfer events in chemical reactions [1–7] and in biological systems, including photosynthetic complexes [8–16] and retinal-based pigments such as halorhodopsins [17,18], bacteriorhodopsins [19–24] and rhodopsins [25,26]. For a given photoreaction, the accessible parameters include the molecular volume change secondary to conformational change or electrostriction, and enthalpy and entropy changes. In addition, photoacoustics may provide information on kinetics and energy efficiencies in chemical systems, with applications ranging from nanomaterials in solar energy storage [27–29] to image analysis in biomedicine [30–32].

To fully understand a chemical reaction, it is necessary to understand not just its kinetics, but also its thermodynamic parameters. Specifically, thermodynamic parameters such as volume change, the free energy change (redox potential of cofactors *in situ*), enthalpy change, and entropy change are crucial elements required to elucidate the mechanism of an electron transfer reaction at the molecular level. However, in contrast to the kinetics of electron transfer mechanisms, thermodynamic information is far less accessible. Thermodynamics reveals the energy levels of reactants and products, as well as the driving forces in a chemical reaction. The driving force of the chemical reaction is the Gibbs free energy, which is composed of enthalpic and entropic components. The theory of electron transfer in chemical and biological system was developed by Marcus [33] and played a key role in advancing the understanding electron transfer mechanisms, including photosynthetic systems [34–37]. However, one weakness of the theory is the omission of entropic contribution in the electron transfer steps [5,38–41].

Pulsed photoacoustics provides novel insights on the entropic contribution to electron transfer in proteins and is able to probe the role of the membrane environment and cofactors *in vitro* and *in vivo* in terms of a broadly thermodynamic view. These unique features of photoacoutstics studies provide the potential for a deeper understanding of the mechanisms of electron transfer and proton-coupled electron transfer in chemical and biological systems. They also provide a framework to modify and improve the existing Marcus electron transfer theory as well as the formulation of a comprehensive electron transfer theory. The results of photoacoustics experiments also provide direct information about the function of membrane proteins central to photosynthesis and vision. In particular, the photosynthetic membrane proteins are excellent models for elucidating electron transfer and proton transfer mechanism in other membrane proteins.

In this review we discussed recent advances in the methodology of pulsed time-resolved photoacoustics and its application to studies of photosynthetic reaction centers and membrane receptors such as the G protein-coupled receptor rhodopsin. Photoacoustics is a unique probe of these two prominent classes of biological photoreceptors, which are organic light sensors and potentially be utilized to sense the cell signaling and protein network in biotechnology and medicine [42].

## 2. Principle of Pulsed Photoacoustics

### 2.1. Molecular Volume Change by Electrostriction and Structural Conformational Changes

**Figure 1.**
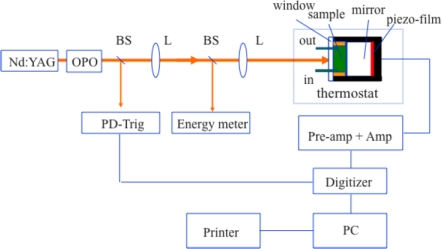
Block diagram of pulsed photoacoustic system on the nanosecond-microsecond time scale. BS = beam splitter, L = lens, Amp = amplifier, OPO = optical parametric oscillator.

Figure 1 shows the experimental setup of the pulsed photoacoustic apparatus, which enables one to determine the volume change and enthalpy of photoreactions on the nanosecond to microsecond time scales. A Nd:YAG laser and OPO are used to produce light of 680 nm and 700 nm with 1-mm light path. Consider the different response time of film, the PA detector containing a 128 μm piezoelectric film and 1-cm thickness of cell are used for the microsecond time scale photoacoustic experiments. A 28 μm film and 1-mm path cell are used for the nanosecond measurements. A 5-cm dielectric mirror was used according to the design of Arnaut *et al*. [43].

The advantage of using the mirror in the photoacoustic system is two-fold: (1) to increase light absorption and (2) to generate the time delay to eliminate the electric artifact in photoacoustic measurements. The volume change or heat of a photochemical reaction generates a pressure in a photoacoustic cell. The pressure is sensed by a piezo film or a microphone and recorded as a form of sound wave, designed photoaoustic wave or photoacoustic signal. To calibrate the photoacoustic signal, a reference must be used. A photoacoustic reference is a sample that degrades all the absorbed energy to heat within the resolving time interval of the apparatus. Two kinds of photoacoustic references are used to ensure the accuracy of the experimental data. The first is an external black ink reference, which is the supernatant fraction of high-quality commercial black carbon ink suspension after centrifugation. The other is an internal reference—the light-saturated photosynthetic material. The photoacoustic signal generated by a reference is described by Equation 1:

(1) $${\mathrm{PA}}_{\mathrm{ref}}=\frac{F\cdot \alpha \prime }{\kappa }E_{a}\cdot I(t)$$

where *E_a_* is photon energy absorbed by the sample, *F* is piezo film sensitivity, α′ is an abbreviation for thermal expansivity/heat capacity × density, *κ* is compressibility, and *I*(*t*) is the impulse response of the system.

The photoacoustic signal is produced by the active photosynthetic reaction centers, which form a charge-separated radical pair upon light excitation, or by the visual receptor proteins, which undergo light-induced structural conformational change. The signal typically consists of two components (Equation 2).

(2) $${\mathrm{PA}}_{\mathrm{RC}}=\frac{F}{\kappa }\left[\alpha \prime \cdot Q_{\mathrm{RC}}+\Delta V_{\mathrm{RC}}\right]\cdot I(t)$$

where *Q_RC_* is the heat output, which includes the enthalpy change of the reaction and other rapidly released heat, and *ΔV_RC_* is the molecular volume change of the reaction.

When the temperature of the sample in aqueous solution is above the maximum density temperature (∼4 °C), the heat output (*Q_RC_*) of a photochemical reaction induces the volume expansion of solution. When the temperature is below ∼4 °C, the heat induces the volume contraction. At ∼4 °C, the heat component (*Q_RC_*) is zero as the expansibility of water is zero. The photoacoustic signal at ∼4 °C is simply the molecular volume change *(ΔV_RC_*). To determine the precise values of the volume change resulting from charge separation in the photosynthetic materials, two different approaches are used: (1) volume yield measurements, and (2) saturation measurements.

In the first approach, the flash yield *(ΔV/E*) is the volume change per unit photon energy absorbed by the system. The flash yield of the photoacoustic *(ΔV/E*) signal is dependent on the excitation photon flux, following Equation (3):

(3) $$\frac{\Delta V_{\mathrm{RC}}}{E}=N\Delta V_{Y}\frac{\left(1-e^{-\Phi \sigma E}\right)}{N\sigma E}$$

where *N* is the number of reaction centers in the illuminated volume of the cell, *ΔV_Y_* is the volume change per reaction center, Φ is the quantum yield of photoreaction, and σ is the optical cross-section per reaction center at the excitation wavelength.

Assuming *ΔV* does not change over a small temperature range, *ΔV_RC_* is obtained in the limiting low pulse energy region (the linear region of Equation 3) by normalizing to the reference photoacoustic signal, converting PA_ref_ to volume *via* α′ at 25 °C and correcting for the change in the compressibility of water between T_m_ and 25 °C:

(4) $$\Delta V_{\mathrm{RC}}=\frac{{\mathrm{PA}}_{\mathrm{RC}}^{Tm}}{{\mathrm{PA}}_{\mathrm{ref}}^{25}}\times \frac{\kappa^{Tm}}{\kappa^{25}}\times \Delta V_{\mathrm{ref}}$$

where *ΔV_ref_* = α′E, is the thermal volume change of the reference at its temperature. Since the system is linear, one can calculate *ΔV_ref_*, the thermal volume change at 25 °C, for each absorbed photon at the excitation wavelength. At low energy, one obtains the volume per center multiplied by the quantum yield, Φ·ΔV_y_. However, if the energy is too low, the photoacoustic signal-to-noise ratio is poor. In the case of photosynthetic reaction centers the value of volume change can be obtained by fitting a curve to Equation 3 and extrapolating to zero excitation energy.

In the second approach, every photosynthetic reaction center is excited to obtain the maximum photoacoustic signal. Fitting of the photoacoustic data to Equation 5 allows one to obtain the light saturated volume change, *ΔV_s_*:

(5) $$\Delta V=N\Delta V_{S}(1-e^{-\Phi \sigma E})$$

where *ΔV_S_* is the volume change produced per photosynthetic reaction center; *σ* is the optical cross section per photosynthetic reaction center; *Φ* is the quantum yield of the photochemical reaction, and *E* is the excitation photon flux.

In this method, one must calculate the number of photosynthetic reaction centers in the illuminated volume of the cell (∼0.34 mL), *N*, to obtain the real volume change *ΔV_s_*. The effective cross-section (*Φ·σ*) can also be obtained from the fit of the curves. This approach measures the absolute number of photosynthetic centers, calculated without assumptions of the quantum yield assuming all centers are successfully “hit” with enough energy. Although these two methods use the same set of data, the calculation of *ΔV* is differently weighted and completely different.

### 2.2. Quantum Yield of Photoreaction

The photoacoustic measurement includes the enthalpy or volume changes times the quantum yield, which can be determined using the light saturation curve of photoacoustics. The light saturation function at 4 °C (where there is no thermal signal) contains the photochemical quantum yield. For a simple system with one cross section, the photoacoustic signal is described by the cumulative one-hit Poisson distribution (Equation 6).

(6) $$\mathrm{PA}=N\cdot {\mathrm{PA}}_{0}(1-e^{-\Phi \cdot \sigma \cdot E})$$

where *N* is the numbers of centers in the sample, *PA_0_* is the photoacoustic signal produced per successful hit of the reaction centers; *σ* and *Φσ* are the optical cross section and effective optical cross section; *Φ* is the quantum yield, *E* is the photon energy absorbed by the sample, and *A* is the absorbancy of solution.

The effective cross section can be obtained by the curve fit of the photoacoustic saturation curve. As the optical cross section, *σ* with units of area per reaction center, can be calculated from the chlorophyll content and ratio of chlorophyll to the primary electron donor, the quantum yield of chemistry can be determined.

### 2.3. Enthalpy Change and Entropy Change of Photoreaction

The enthalpy change of electron transfer reactions in photosynthetic reaction centers and photoreceptors can be calculated by the Equation 7:

(7) $$\Delta H=(E_{h\nu }-E_{\mathrm{trap}})-\frac{\left(\frac{{d\left(\mathrm{PA}\cdot \kappa \right)}_{\mathrm{RC}}}{d\alpha }\right)}{\left(\frac{{d\left(\mathrm{PA}\cdot \kappa \right)}_{\mathrm{ref}}}{d\alpha }\right)}\times E_{h\nu }$$

As discussed above, pulsed photoacoustics directly measures the enthalpy and volume changes of the reactions on the nanosecond and microsecond time scales. With the given free energy, the entropy change is calculated from *ΔG = ΔH* − *TΔS*.

### 2.4. Measuring the Reaction Enthalpy and Volume Changes

**Figure 2.**
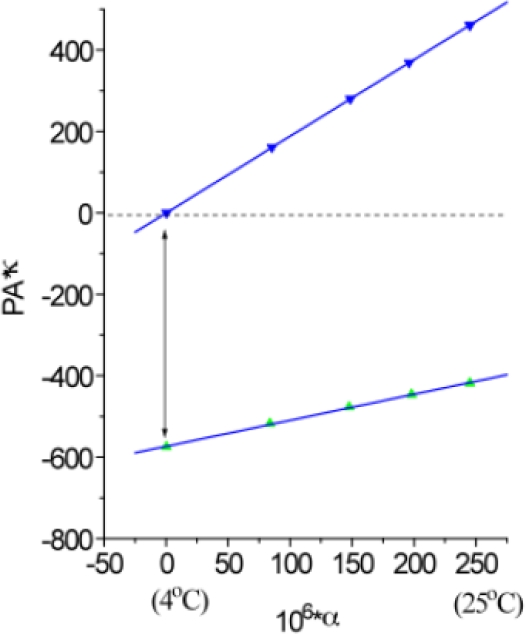
Typical plot of the product of the photoacoustic signal and water compressibility (κ) versus water expansivity (α) for ink reference (blue symbols) and PS I complex (green symbols).

To determine the reaction enthalpy and volume changes, the photoacoustic waves of ink references and photoreceptor samples such as PS I complexes are recorded at five temperatures, 4, 10, 15, 20, and 25 °C. A typical data analysis is plotted in the product of the photoacoustic intensity (peak to peak value) and water compressibility versus the water expansivity (Figure 2). The linear fit of the experimental points gives intercept and slope of ink reference and PS I samples, respectively. The molecular volume change of PS I on the 1 μs time scale can be easily obtained by inserting the intercepts values of the linear fit of ink reference and PS I sample to the Equation 4. The enthalpy change of the photochemical reaction in PS I on the 1 μs time scale may be found *via* the Equation 7 using the slopes of the fit.

The deconvolution analysis on photoacoustic waves allows one to time-resolve the thermodynamic parameters of each individual step of photoreaction [2,39,44]. The photoacoustic pressure wave is caused by the rate of the heat release. The two or more steps of a photochemical reaction will change the shape (wider or narrower) of photoacoustic wave than the photoacoustic reference.

*PA_s_(t)* is convoluted (*) with the time derivative, h_q_’(t), of the assumed heat and volume change function h_q_(t). For convenience, the amplitute of the photoacoustic signal may be separated into three fractions:

(8) $${\mathrm{PA}}_{s}\;(t)=A_{f}\;{\mathrm{PA}}_{\mathrm{ref}}\;(t)+A_{s}\;{\mathrm{PA}}_{\mathrm{ref}}*h_{q}{}^{\prime }\;(t)+A_{r}\;0$$

where *PA_s_(t)* and *PA_ref_(t)* is the time functions of photoacoustic wave of a sample and reference, respectively. *A_f_* is the fraction of the total energy absorbed, which is released rapidly. The second term describes the fraction of a slow rat in the measurable time domain (*A_s_*). The fraction of the residual heat release and volume changes stored in the system for a long time is denoted A_r_. As the photoacoustic signal is weight by the rate of reaction, the fraction of residual component is small and not assessable within the measurable time window.

For instance, the deconvolution fit of the photoacoustic wave of PS I using Equation 8 may yield the amplitudes of a prompt component (*A_f_*) on the nanosecond time scale and of a slow component (*A_s_*) on the microsecond time scale, respectively. A plot of *A_f_* and *A_s_* obtained by deconvolution over the whole temperature range may be constructed in the same way as shown in Figure 2. The slope of the plot enables one to calculate the enthalpy change for the each individual step of the photoreaction, and the intercept of the plot gives the volume change.

## 3. Photoacoustic Studies of Photosystems

### 3.1. Structure and Function of Photosystems

**Figure 3.**
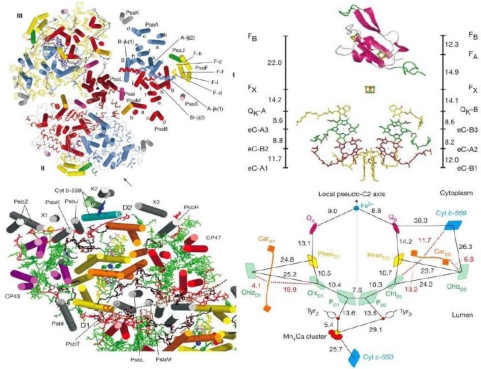
Structures and arrangement of cofactors in PS I (upper) [46] and PS II (lower) (Reproduced with permission from Nature publishing Group [47]).

The three-dimensional structures of both Type I [45,46] and Type II [47,48] photosynthetic reaction centers have been reported. Figure 3 shows the structures and arrangement of cofactors of cyanobacterial photosystems I (PS I) at 2.5 Å resolution [46] and of cyanobacterial PS II at 3.0 Å resolution [47].

**Figure 4.**
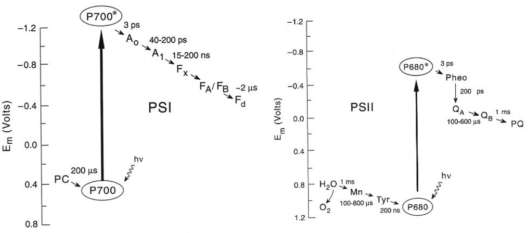
Pathways and rates of electron transfer reactions in PS I (left panel) and PS II (right panel).

These structures reveal the binding sites and precise orientation of cofactors and their interaction with proteins and provide a solid basis to interpret results of photoacoustic studies at an atomic level. In addition to high-resolution structural information, the kinetics and thermodynamics of electron transfer reactions in biological systems are important for understanding precise mechanisms. During the past decade the kinetics of electron transfer steps in reaction centers of anoxygenic and oxygenic photosynthesis has been thoroughly investigated over the timescale of femtosecond to second [49–52] as shown in Figure 4. However, the thermodynamics of electron transfer steps in photosynthesis, such as volume change, enthalpy and entropy, is far less well understood. There are at least two reasons to measure these thermodynamic parameters accurately. First, knowledge of the thermodynamic parameters of electron transfer reactions allows one to gauge the efficiency of energy conversion, Efficiency refers to the amount of the solar energy stored in the photosynthetic organisms. The second motivation for investigating thermodynamics is to separate the free energy into its enthalpy and entropy components, which provides more details and deeper understanding of the driving force of electron transfer mechanisms.

The pulsed photoacoustic technique gives a direct measurement of the enthalpy change of photosynthetic reactions [38,39]. A microphone may detect the photoacoustic waves *via* the thermal expansion in the gas phase. The 50-fold larger thermal expansion of a gas over liquid makes the microphone more sensitive. A gas-coupled microphone in a closed chamber is used as a detector on the ms time scale. This method allows *in vivo* measurements of the photosynthetic thermal efficiency, or energy storage, and of the optical cross-section of the light harvesting systems [3,53,54]. However, the time resolution is low, typically about 30 μs, and inappropriate for obtain thermodynamic parameters on the microsecond and nanosecond time scales. The use of piezoelectric films acoustically coupled to a liquid sample and a pulsed laser light source increased the time resolution of the photoacoustic technique to the microsecond scale [55,56]. Photoacoustic thermodynamic studies have been carried out on isolated photosynthetic reaction centers from bacteria *Rb. sphaeroides* [57], on PS I from cyanobacteria [58], and on PS II from spinach and *Chlamydomonas reinhardtii* [58].

Measuring the energetics of photobiological reactions *in vivo* is of more interest because the local environment of photoreceptors may exert a prevailing consequence on their kinetics and thermodynamics. Pulsed photoacoustics *via* a microphone detector revealed the oxygen evolution and oxygen uptake *in vivo* on the millisecond time scale [53]. Using a piezo film detector, the *in vivo* enthalpy and volume changes of photosystem I and photosystem II of *Synechocysits sp. PCC 6803* were obtained on the microsecond time scale [61]. The contribution of the two photosystems was distinguished by excitation at two different wavelengths, 625 nm for predominant excitation of PS I and 680 nm for PS II, respectively. The difficulty in the photoacoustic measurements of intact cells is the heterogeneous or “cell” artifact at the temperature of maximum density of near 4 °C. To correct the “cell” artifact, five measurements are needed instead of usual three (see reference [61] for details). The enthalpy and volume changes of intact cells of *Synechocystis sp. PCC 6803* were in good agreement with those of isolated complexes within the experimental errors.

Theoretical calculations of electron transfer have often assumed the reaction entropy to be zero. For example, the standard formulation of Marcus theory assumes that the vibrations coupled to electron transfer have the same frequency in the reactant and product states [33]. Marcus theory introduces reorganization energy to interpret the reaction rate of electron transfer reactions. The reorganization energy can have two contributions: a vibration term and a solvent term. Treatments of the temperature dependence of the rate of electron transfer often assume that the free energy is independent of temperature [59]. However, these assumptions are called into question by recent work. For instance, the entropy change often neglected in an artificial photosynthetic system was actually determined to be large [11]. A fit of Marcus reorganization energy cannot interpret the observed volume change of electron transfer reaction.

### 3.2. Photoacoustic Studies of Photosystems

**Figure 5.**
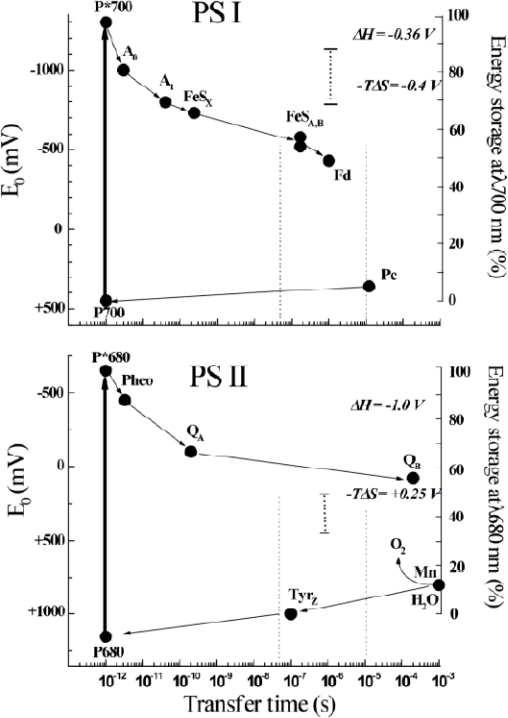
Energetic scheme of PS I and PS II. Dashed lines show the time window of the PA measurements (reproduced with permission from the American Chemical Society [62]).

Using pulsed photoacoustics, the volume change and enthalpy of electron transfer reaction were measured in aqueous solution [39] and in the photosynthetic reaction center complex of *Rb. sphaeroides* [38]. A large entropy was calculated based on these measurements. Further photoacoustic measurements revealed that the entropy change of electron transfer in PS I trimer from *Synechocystis* sp. PCC 6803 on the microsecond time scale was the same as that in bacterial centers [60]. The volume contraction of reaction centers of PS I, which results directly from the light-induced charge separation forming P_700_^+^F_A_*/*F_B_^−^ from the excited state P_700_*, was determined to be −26 Å^3^. The enthalpy of the above electron transfer reaction was found to be −0.39 eV. Taking the free energy of the above reaction as the difference of their redox potentials *in situ* allows one to calculate an apparent entropy change (*TΔS*) of +0.35 eV. In contrast, electron transfer in PS II core complexes from *Synechocystis* sp. PCC 6803 is accompanied by a small negative entropy change [61]. At pH 6.0, the volume contraction of PS II was determined to be −9 Å^3^, and the enthalpy change −0.9 eV for the formation of the state P_680_^+^Q_A_^−^ from P_680_*. The ΔV of PS II, smaller than that of PS I and bacterial centers, is assigned to electrostriction and analyzed using the Drude-Nernst equation. To explain the small ΔV for the formation of P_680_^+^Q_A_^−^ or Y_Z_^•^Q_A_^−^ we proposed that fast proton transfer into a polar region is involved in this reaction. These observations were confirmed using intact living cells of the same organism [62]. These thermodynamic parameters are summarized in Figure 5. The enthalpies for the formation of states P_700_^+^F_AB_^−^ from P_700_* and Y_Z_^•^P_680_Q_A_^−^ from P_680_* *in vivo* were estimated to be about −0.3 eV and −1 eV, respectively. Comparison of these values with the free energies of the reactions indicates a significant contribution of the apparent entropy changes *TΔS*, +0.4 and −0.24 eV for the formation of ion-radical pairs in PS I and PS II, respectively.

To understand more details of electron transfer thermodynamics, it is of interesting to determine the thermodynamic parameters of electron transfer in the photosynthetic electron transfer mutants on both the nanosecond and microsecond time scales. It has been generally believed that phylloquinone is difficult to dislodge from the A_1_ binding site in PS I, in contrast to the ubiquinone (Q_A_) in bacterial centers from *Rb. Sphaeroides* that can be easily replaced by a wide variety of different quinones [59,63]. A biological method to remove phylloquinone was recently devised. Targeted inactivation of the *menA* and *menB* genes that code for phytyl transferase and napthoate synthase in the phylloquinone biosynthetic pathway [64] precludes its availability for incorporation in the A_1_ site. Yet, in spite of the demonstrated absence of phylloquinone, the *menA* and *menB* null mutants grow photosynthetically. EPR measurements show that plastoquinone-9 (A_P_) is recruited into the A_1_ site [64] and functions as an efficient 1-electron electron carrier [65]. Time-resolved optical studies indicate the forward electron transfer from A_1_^−^ to F_X_ is slowed 1000-fold, to 15 and 300 μs, compared to 20 and 200 ns in wild-type PS I [66]. Given the altered kinetics of electron transfer, it will be of interest to investigate the effect of these mutations on the thermodynamics of electron transfer in PS I.

These thermodynamic parameters reveal that the driving force in the photosynthetic reactions may be both enthalpic and entropic. However, most of thermodynamic parameters using photoacoustics are on the 1-μs time scale. In order to obtain detailed information on intermediates in the PS I reactions, we measured the volume change and enthalpy change on the nanosecond time scale. The time constant of charge transfer from A_1_^−^ to F_A/B_ is reported to be 20 to 200 ns [51]. However the modeling analysis of the electron transfer reactions in PS I by electron tunneling theory indicate complex equilibria between the various species [67]. The enthalpy and volume changes associated with this reaction and with the charge separation of the P_700_* → A_1_ reaction seem unknown so we investigated these reactions by fast pulsed photoacoustics on the nanosecond time scale. Unexpectedly and strikingly, a larger enthalpy change was observed by the peak-to-peak PA analysis on this time scale than that observed on the μs time scale.

**Figure 6.**
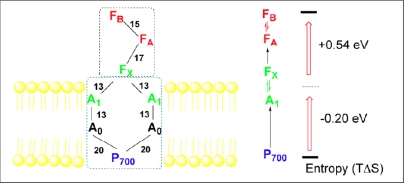
Thermodynamic and kinetic assignments of photoacoustic measurements in PS I of *Synechocystis* sp. PCC 6803 (reproduced with permission from the American Chemical Society [40]).

The photoacoustic waves of PS I involved the contributions of the initial and subsequent electron transfer reactions (Figure 4). As shown in Figure 6, deconvolution analysis of photoacoustic signals on nanosecond and microsecond time scales resolved enthalpy and volume changes of two steps and produced a positive enthalpy change for the second step. The fit by deconvolution of photoacoustic waves on the nanosecond and microsecond time scales resolved two kinetic components: (1) a prompt component (<10 ns) with large negative enthalpy change (−0.8 ± 0.1 eV) and large volume change (−23 ± 2 A^3^), which are assigned to the P_700_* → A_1_^−^F_X_ step, and (2) a component with ∼200 ns lifetime, which has a positive enthalpy change (+0.4 ± 0.2 eV) and a small volume change (−3 ± 2 A^3^) which are attributed to the A_1_^−^F_X_ → F_A/B_^−^ step. For the fast reaction using the redox potentials of A_1_F_X_ (−0.67 V) and P_700_ (+0.45 V) and the energy of P_700_* (1.77 eV), the free energy change for the P_700_* → A_1_^−^F_X_ step is −0.63 eV. Thus, the entropy change (*TΔS*, *T* = 25 °C) is −0.2 ± 0.3 eV. For the slow reaction, A_1_^−^F_X_ → F_A/B_^−^, taking the free energy of −0.14 eV [67], the entropy change (*TΔS*) is positive, (+0.54 ± 0.3 eV).

The redox thermodynamics of many FeS proteins (ferrodoxins) have been determined. Interestingly, most of the Fe_4_S_4_(Cys)_4_ proteins have positive enthalpies of reduction of +0.3 to +0.4 eV, while the others have very small negative enthalpies. The authors explain the enthalpies in terms of electrostatic interactions with the protein dipoles. All the reduction entropies are negative as anticipated from the increase in charge and seem to vary in magnitude opposite to the enthalpies. Thus the positive enthalpy of the A_1_^−^F_X_ to F_A/B_ reaction may be assigned in part to the FeS cluster reduction. The positive entropy may have contributions from the freeing of oriented polar groups on quinone anion oxidation. The previous step has negative entropy as expected.

**Figure 7.**
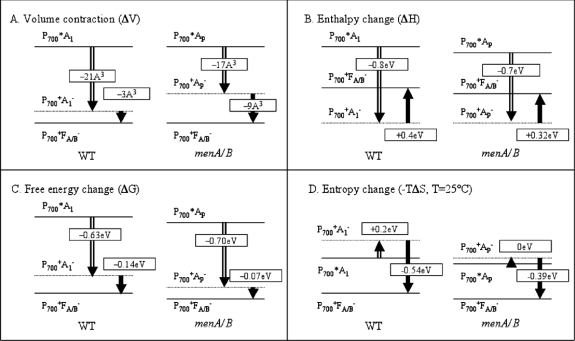
Thermodynamic parameters of charge separation in *menA/B* PS I and wild-type PS I from *Synechocystis* 6803 (reproduced with permission from the American Chemical Society [8]).

To summarize the thermodynamic data, we present the volume changes, free energies, enthalpy and entropy changes on *menA/B* PS I in comparison with those on the wild-type PS I in Figure 7. Opened arrows are the early step forming P_700_^+^A_1_^−^ from P_700_* for the wild-type PS I or P_700_^+^A_P_^−^ from P_700_* for the mutants, and solid arrows are the number of the following reaction: P_700_^+^A_P_^−^ → P_700_^+^F_A/B_^−^. As shown in Panel A, the volume contraction of early step of photoreaction in the mutants (−17 Å^3^) is smaller than that in the wild type (−21 Å^3^). Similarly, the enthalpy change (−0.7 eV) of the early step in the mutants is smaller than that (−0.8 eV) in wild type PS I (Figure 7A and 7B).

Assuming a redox potential of −0.6 V for plastoquinone-9 in the A_1_ site [66], the free energy (−0.7 eV) of this early reaction in the mutants is larger than the value (−0.6 eV) in the wild type as indicated in Figure 7C. Taking the difference of free energy and enthalpy change in the mutants, the apparent entropy change of the early step in mutants is zero. In contrast, the apparent entropy change in the wild-type PS I is calculated to be +0.2 eV. Since the apparent entropy change for the overall reaction of the generation of P_700_^+^ F_A/B_^−^ from P_700_^*^ is +0.35 eV [60], it implies that the latter reaction in the mutants, *i.e.*, the P_700_^+^ A_P_^−^ F_A/B_ → P_700_^+^ A_P_ F_A/B_^−^ reaction, is almost completely entropy-driven (TΔS = +0.4 eV and ΔG= −0.1 eV) (Figure 7D). Therefore, based on our experimental results we propose that the foreign quinone (A_P_) in PS I does affect the thermodynamics of charge separation in the early steps in PS I with a smaller volume and enthalpy changes, a large free energy and zero entropy change. The deconvolution of the time derivative of the volume or heat release function with the apparatus response function provided by the reference signal enables us to resolve the fast and slow photoacoustic components [25,39,68].

The observed thermodynamic data of the *menA* and *menB* null mutants show little difference. This is expected because the recruited quinone (A_P_) is the same in both mutants. Volume change of mutant PS I following charge separation on both time scales is −16 ± 2 Å^3^. The quantum yield of charge separation in PS I of the mutants is slightly lower (85 ± 10%) than that of the wild-type PS I (96 ± 10%). The observed reaction is assigned to the formation of P_700_^+^A_P_^−^ F_A/B_ from P_700_*A_P_ F_A/B_. The enthalpy change (ΔH) of about −0.69 ± 0.1 eV in mutant PS I was obtained for this reaction. In contrast, a large enthalpy change of ∼ −1.0 eV for the formation of P_700_^+^A_1_^−^ from P_700_* in the wild-type PS I was observed. These results strongly suggest that not only the kinetics but also the thermodynamics of electron transfer reactions in PS I is significantly affected by the recruitment of the foreign plastoquinone-9 into the A_1_ site [8].

## 4. Photoacoustic Studies of Receptors

### 4.1. Structure and Function of G Protein-Coupled Receptors

Membrane receptors facilitate cell-cell communication and allow cells and organisms to sense their environment. Receptors interact with specific ligands, which range from proteins or peptides, to small molecules, such as neurotransmitters, hormones, pharmaceutical drugs, or toxins. The largest class of receptors in the human genome is the family of G protein-coupled receptors (GPCRs), also known as 7-transmembrane (TM) or heptahelical proteins [69]. In human sensory systems, physiology and pathophysiology, including vision and taste perception, glucose metabolism, the brain’s response to dopamine and the ability of the AIDS virus to enter human cells, GPCRs play a central role [70].

**Figure 8.**
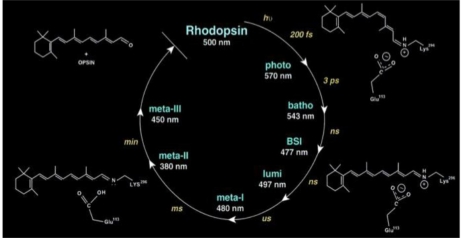
Photoreaction of bovine rhodopsin (reproduced with permission from the American Physiological Society [71]).

Rhodopsin, a highly specialized integral membrane protein, is the most extensively studied member of the GPCR family. It detects photons in the rod photoreceptor cell responsible for dim-light vision. Rhodopsin is the first GPCR whose crystal structure has been reported at high resolution [72]. The rhodopsin structure provides a blueprint to explore and understand the mechanism of rhodopsin activation, which should also provide insights into the general mechanism of activation of other GPCRs [71]. Recent reports of crystal structures of additional GPCRs and of a ligand-free opsin now provide an opportunity to compare and contrast modes of ligand binding and G protein activation among the entire superfamily of heptahelical receptors [73–75]. Rhodopsin has three topological domains: the extracellular surface, the membrane-embedded domain, and the intracellular surface. The amino terminus of rhodopsin is extracellular; the carboxy terminus is intracellular. The 11-*cis*-retinal chromophore in rhodopsin is linked to the protein through a protonated Schiff base (PSB) whose positive charge is neutralized by the Glu-113 counterion [76–78]. The photocycle of bovine rhodopsin is shown in Figure 8. After absorption of a photon, the 11-*cis*-retinal chromophore is isomerized with high quantum efficiency, leading to intramolecular proton transfer and significant protein conformational changes. The isomerization event triggers the photoactivation process and results in the formation of active species, metarhodopsin II (Meta II), which binds the heterotrimeric G protein, transducin.

### 4.2. Photoacoustic Studies of Rhodopsins

The enthalpy and volume changes taking place during the stepwise photoinduced transformation of rhodopsin were investigated by pulsed photoacoustics [26,79–82]. The quantum yield of the highly efficient photoreaction to produce the first photointermediate, batho, within <1 ps in rhodopsin is 0.67 [83]. The energy stored in the batho intermediate is estimated to be between 109 (1.14 eV) and 146 kJ/mol (1.52 eV) [84], which is about 60% of the absorbed photon energy. Using detergent solubilized rhodopsin a total structural volume change of 29 mL/mol for the transition of rhodopsin to lumi and a corresponding enthalpy change of 227 kJ/mol were obtained [85].

**Figure 9.**
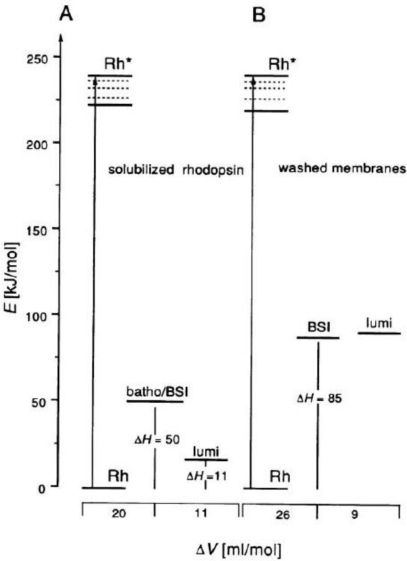
Energy levels of the intermediates and the structural volume changes for the photoinduced transformation of rhodopsins (reproduced with permission from Elsevier [25]).

Using a detailed kinetic analysis and a deconvolution procedure, the volume and enthalpy changes of photoreactions in solubilized bovine rhodopsins and washed retinal rode outer (ROS) segment membranes were determined in the time range from 20 ns to several microseconds in the absence and presence of detergent dodecyl-β-d-maltoside as shown in Figure 9 [25,26]. The energy content of BSI is similar to that of lumi, 85 kJ/mol (0.88 eV). The volume changes are an expansion of 26 mL/mol (16 A^3^ per molecule) for the transition rhodopsin to BSI, and a further expansion of 9 mL/mol (5.4 A^3^ per molecule) for the BSI to lumi transition. As the energy levels of BSI and lumi are nearly the same, entropy should control the exergonic reaction. The comparison of the thermodynamic parameters for the batho to BSI and the BSI to lumi transformations in intact membranes and in solubilized rhodopsin implies that both transitions break and reform the weak interactions around regions of the protein. The total volume change for the rhodopsin to lumi is 31–35 mL/mol (19–21 A^3^ per molecule).

**Figure 10.**
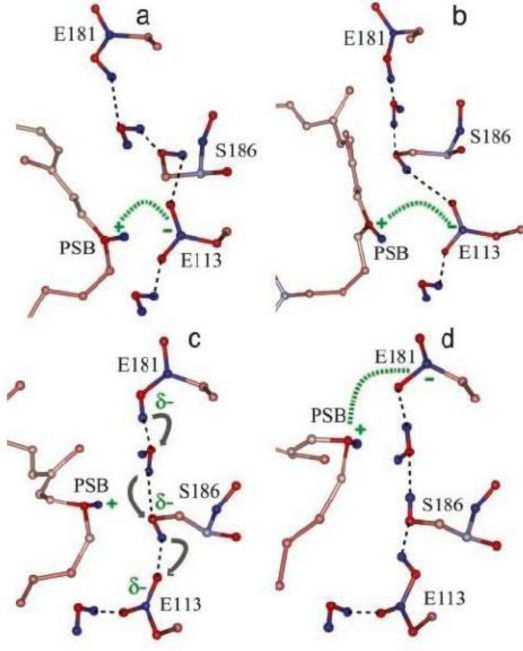
Counterion switch in rhodopsin photoactivation (reproduced with permission from the National Academy of Sciences [88]).

The crystal structure of bovine rhodopsin at high resolution [72] surprisingly revealed that Glu-181 in extracellular loop II is only 4.7 Å from C_12_ of 11-*cis*-retinal. Glu-181 may play a vital role of photoactivation of rhodopsin [86,87]. For example, Glu-181 might be able to exert significant electrostatic interaction on the chromophore. Preresonance Raman vibrational spectra of the unphotolyzed E181Q mutant are nearly identical to spectra of the native rhodopsin, supporting that Glu-181 is uncharged or protonated in the dark state [87]. The pH dependence of the absorption feature of Meta I-like photoproduct revealed a dramatic shift of its Schiff base pKa. This result indicated that Glu-181 is the primary counterion switch of the retinylidene prontonated Schiff base in the Meta I state. Figure 10 shows the possible mechanism of counterion switch in rhodopsin in Meta I state [88].

According to this hypothetical model, two water molecules and Ser-186 form an H-bond chain between Glu-113 and Glu-181. Glu-113 is the primary PSB counterion in the dark state. After photolysis, the chromophore isomerizes to form batho and then the blue-shift intermediate (BSI) state. The two water molecules and Ser-186 line up to prepare for the proton transfer and PSB shift relative to Glu-113. In the lumi state, the PSB shifts further away from Glu-113 toward Glu-181. The proton transfer is completed in the Meta I state. The PSB group is now close to Glu-181 forming a new counterion.

The proposed counterion switch of E181 was demonstrated to play a key role in photoactivation of rhodopsin [88–90], and the E181Q mutant rhodopsin showed an altered kinetics of photoreaction [91]. However, the effect on thermodynamics is unknown. Using pulsed photoacoustics we are able to determine the volume change and enthalpy of photoreactions in E181Q mutant rhodopsins on the nanosecond and microsecond time scales. Preliminary photoacoustic data showed that the volume changes in E181Q mutant and wild-type rhodopsin are +72 ± 7 and +37 ± 4 Å^3^, respectively. Our estimate of electrostrictive changes caused by movement of the charges is smaller and of opposite sign. Thus, most of the change must be a conformational volume change, similarly to what has been observed in bacteriorhodopsin. The significant difference in conformational change between E181Q and native rhodopsin supports a functional role for Glu181 in early steps of rhodopsin photoactivation.

## 5. Conclusions

### 5.1. Potential Problems and Future Efforts

The thermodynamic parameters of electron transfer in photosynthetic reaction center complexes and in whole cells of *Synechocystis* have been determined using pulsed photoacoustics [60–62]. However, the parameters in *Synechocystis* PS II were inconsistent with those obtained using *Chlamydomonas* and spinach chloroplast [58,92]). The *in vitro* measurements, in particular, showed a significant difference from the *in vivo* values. These discrepancies in purified complexes from different organisms such as *Synechocystis*, *Chlamydomonas*, and spinach chloroplasts must be clarified.

We previously determined the volume changes and enthalpy changes of electron transfer in *Synechocystis* PS I complexes on the microsecond time scale [60] and then time-resolved two individual steps in the complexes on the nanosecond time scale [40]. A significant entropic component was found. In future work, we intend to determine the thermodynamic parameters in PS I *in vitro* and *in vivo* using photosynthetic electron transfer mutants to uncover the roles of the protein matrix in electron transfer steps. In contrast, for the volume changes in PS I and bacterial reaction centers with an reasonable number within the error margins, the volume change of PS II charge separation is in the range from −2 to −16 Å^3^ [60]. For example, Boichenko *et al.* [61] reported a −2 Å^3^ in the whole cells of cyanobacterium *Synechocystis*, and Delosme *et al.* [13] observed −9 Å^3^ from *Chlamydomonas* to −16 Å^3^ from spinach. This obvious inconsistency needs to be investigated and clarified. The pH, species of organisms, redox potentials of electron donor/acceptors, and cell artifacts in the photoacoustic measurements may cause the discrepancy.

The discrepancy might arise from the fact that the measurements were performed at different pH values. The volume change of our previous photoacoustic measurements showed a strong pH dependence of volume change in PS II core complexes. A volume change of −3 Å^3^ was observed at pH 9.0 and −9 Å^3^ at pH 6.0 [61]. Future work may determine the volume change of charge separation in PS II complexes using our previous photoacoustic procedures over a range of pH. The volume change will be measured by the yield and saturation method, respectively. As Delosme et al. pointed out, the discrepancy of the volume change may be due to the use of different organisms: cyanobacteria *versus* green plants [13]. We will test this possibility by using the photosystem II preparations from cyanobacteria, green algae, and higher plants. These PS II preparations can be purified from cyanobacterium *Synechocystis* 6803, from green algae *Chlamydomonas reinhardii*, and from spinach chloroplasts following our established method [93]. The volume change of charge separation in PS II should be determined and evaluated against the data from different groups.

The inconsistency in volume changes and enthalpies of electron transfer could be due to the difference in intactness: *in vitro versus in vivo*. Most of the *in vitro* preparations gave around −10 Å^3^ compared to −2 Å^3^ for the *in vivo* system. A number of factors *in vivo* are different from the *in vitro* preparations. The overlap of absorption of PS I and PS II in the whole cell preparations is significant. We estimated 30% PS II contributions and 70% PS I at 625 nm. In contrast, the contribution of PS I is 20% PS I and of PS II is 80% at 690 nm, respectively [62]. Use of the PS I-depleted mutant may be required to address this problem. Based on preliminary unpublished data Delosme *et al.* argued that the thermal efficiency of purified PS II core from *Thermosynechococcus elongatus* strongly depends on the experimental conditions [13], such as the electron donor or acceptor used, and could be significantly higher than our previous data. This discrepancy needs to be clarified.

A number of photosynthetic electron transfer mutants in addition to chemically modified the reaction centers altered the kinetics of photosynthetic reactions. For example, the photosynthetic electron transfer mutants, including *menA*, *menB*, and *menG* null PS I mutants and A_0_ to F_X_, F_A_, and F_B_ mutants, are available. Pulsed photoacoustics may probe these mutants to explore the effects of mutation on thermodynamic parameters of electron transfer in PS I. The focus should be placed on the effects of the mutations on thermodynamics of photoreactions in photosynthesis. However, the entropic contribution may suffer from the limited published data reporting free energy values. To overcome these obstacles, the electrochemical measurements to determine the redox potentials *in situ* and computational study to calculate the redox potentials of the cofactors will be beneficial. It has been a long-term goal to reveal the thermodynamics of PS II oxygen evolution. However, the time window of current photoacoustics is on the microsecond time scale, which is too fast to determine the millisecond step of PS II oxygen evolution cycle. Recently, using photothermal beam deflection techniques the enthalpies of PS II water oxidation were reported [94]. Using a novel photopressure cell that enables one to obtain the volume and enthalpy changes on the microsecond to second time scales [21], the thermodynamic parameter of reactions involved in PS II oxygen evolution might be determined.

An unexpected discovery in the crystal structure of bovine rhodopsin pointed out the critical role of the extracellular domain in the retinal-binding pocket. The combination of various spectroscopic methods with site-directed mutagenesis was employed to explore the function of the extracellular domain. The difference spectroscopy techniques, such as Fourier-transform infrared (FTIR) and UV-visible difference spectroscopy, make use of the chromophore as an optical switch. Over-expression of recombinant rhodopsin allows a variety of biophysical methods to be used to address particular questions related to protein and chromophore conformational changes. Thermodynamic information revealed by pulsed photoacoustics is supplemental to the structural and dynamic changes in protein conformational change in the vertebrate visual cascade revealed by other methods. For example, pulsed photoacoustics may determine the energy content in the E181Q mutant rhodopsin to uncover more details on the function of E181 in photoactivation of rhodopsins. The preliminary results on volume changes of overall and deconvolution analysis of three steps of photoreaction in E181Q mutant and native rhodopsins revealed significant differences in the photoreaction in these two samples. Future work may refine volume changes and determine enthalpy changes of photoactivation in this important rhodopsin mutant. It is of obvious interest to learn, in addition to the values determined for the fastest steps of the phototransformation, about the heat release and structural volume changes occurring during the binding and activation of the G protein, which takes place during the lifetime of metarhodopsin II in the millisecond time range. For example, photopressure measurements may provide the volume change and enthalpy change of G protein activation.

### 5.2. Concluding Remarks

Knowledge of both the thermodynamics and the kinetics of a reaction are required for its detailed understanding. However, in contrast to kinetics of electron transfer mechanisms, thermodynamic information is far less advanced. Pulsed photoacoustic methodology enables one to extract directly thermodynamic parameters, which include the molecular volume change *via* conformational change or electrostriction and energy levels of photoactivation of receptor and photosystems. Recent results show that some electron transport steps are entropy-driven with protein contribution, the further work on these interesting systems is especially worthwhile. Future work will permit the extraction of enthalpic and entropic contributions to the electron transfer reactions in photosynthetic systems and proton transfer process in visual proteins. The results derived from the project may provide novel insights into the electron transfer and/or proton transfer reaction mechanisms in biological systems in general. As a primary model for transmembrane signaling, the photoacoustic work may provide insights on the driving force of photoactivation of the rhodopsin photoreceptor and have clinical implications for understanding retinal and macular degeneration, night blindness, color blindness and other vision disorders.

## Abbreviations

α’: thermal expansivity/heat capacity × density
ΔG: free energy change
ΔH: enthalpy change
ΔS: entropy change
ΔV_RC_: volume change of the reaction
ΔV_Y_, ΔV_S_: volume changes per reaction center
Φ: quantum yield
κ: compressibility
A: absorbancy of solution
A_1_: quinone electron acceptor of photosystem I
Amp: amplifier
A_P_: plastoquinone acceptor in menA and menB null photosystem I
Batho: bathorhodopsin
BS: beam splitter
BSI: blue-shift intermediate
E181: counter-ion switch in Meta I state in rhodopsin
E_a_: photon energy
F: piezo film sensitivity
F_A_, F_B_, F_X_: iron sulfur clusters of photosystem I
GPCR: G protein-coupled receptor
I(t): impulse response of the system
L: lens
Lumi: lumirhodopsin
*menA*: gene of phytyl transferase in the phylloquinone biosynthetic pathway
*menB*: gene of napthoate synthase in the phylloquinone biosynthetic pathway
Meta I: metarhodopsin I state
Meta II: metarhodopsin II state
N: number of reaction centers
OPO: optical parametric oscillator
P_700_: primary electron donor of photosystem I
P_680_: primary electron donor of photosystem II
PA: photoacoustic
Photo: photorhodopsin
PS: photosystem
Q_A_, Q_B_: quinonoe electron acceptors of photosystem II
Q_RC_: heat output
TM: transmembrane
Y_Z_: tyrosine electron donor of photosystem II
